# Assessment of the Wound-Healing Properties of Almond Oil in a Murine Excisional Wound Model: Biochemical Mechanisms and Translational Perspectives

**DOI:** 10.1021/acsptsci.6c00453

**Published:** 2026-09-30

**Authors:** Alessia Arangia, Regina Aparo, Marika Cordaro, Roberta Fusco, Giuseppina Mandalari, Rosanna Di Paola, Salvatore Cuzzocrea, Ramona D’Amico, Rosalba Siracusa, Daniela Impellizzeri

**Affiliations:** † Dpt. of Chemical, Biological, Pharmaceutical and Environmental Sciences, 18980 University of Messina, 98166 Messina, Italy; ‡ Dpt. of Biomedical, Dental and Morphological and Functional Imaging University of Messina, Consolare Valeria, 98125 Messina, Italy; § Dpt. of Veterinary Sciences, University of Messina, 98168 Messina, Italy; ∥ Dpt. of Pharmacological and Physiological Science, Saint Louis University School of Medicine, Saint Louis, Missouri 63104, United States; ⊥ Link Campus University of Roma, Via Del Casale Di San Pio V 44, 00165 Roma, Italy; # Dpt. of Medicine and Surgery, “Kore”, University of Enna (UKE), 94100 Enna, Italy

**Keywords:** Wound healing, almond oil, skin repair, anti-inflammatory, oxidative stress.

## Abstract

Wound healing is a complex biological process involving overlapping phases of hemostasis, inflammation, proliferation, and remodeling, coordinated by a network of cytokines, growth factors, and cellular responses. Natural products, particularly plant oils, are increasingly being studied for their antioxidant, anti-inflammatory, and regenerative properties. Specifically, this study evaluated the healing potential of almond oil through topical application in an excisional wound model induced in mice, treated once daily for 7 days. Histological, biochemical, and molecular analyses demonstrated that topical treatment with almond oil accelerates wound healing, collagen deposition, and re-epithelialization, and stimulates angiogenesis. At the molecular level, almond oil reduced the expression of MMP-9, increased TGF-β and VEGF, and activated the Nrf2/HO-1 antioxidant pathway, helping limit oxidative and nitrosative stress. Furthermore, it inhibited NF-κB activation and reduced levels of pro-inflammatory cytokines (IL-1β, TNF-α, IL-6). These results indicate that almond oil promotes skin healing through synergistic anti-inflammatory, antioxidant, and regenerative actions, supporting its use as a natural and sustainable topical agent for wound management in clinical settings.

## Graphical abstract

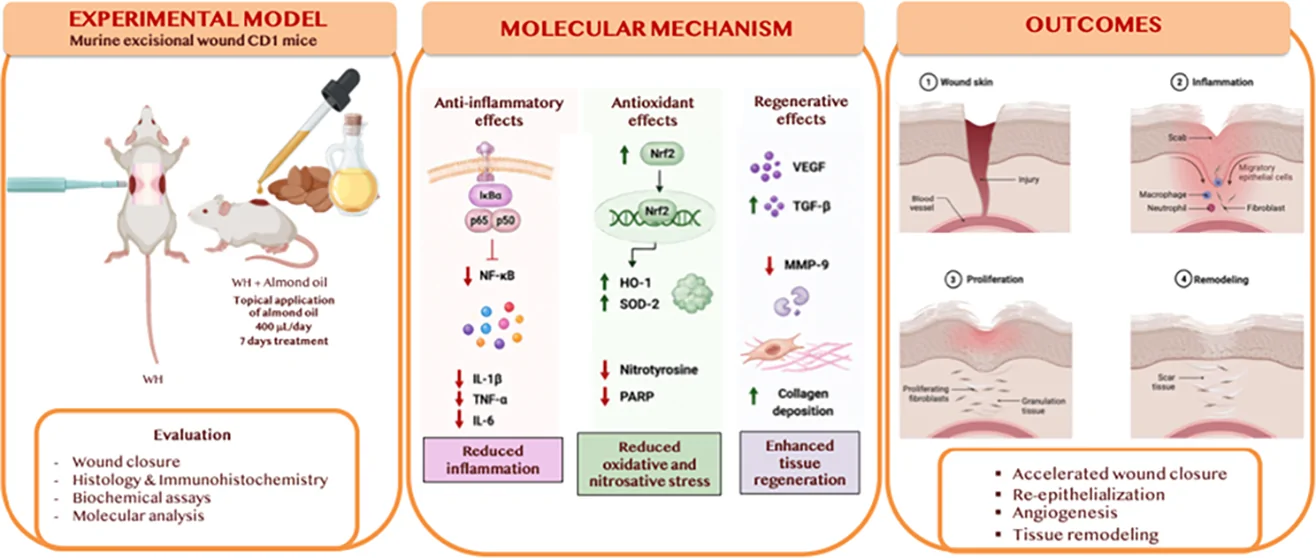

Wound healing (WH) is a complex and finely regulated biological process that restores the structural and functional integrity of damaged tissues.^1^ Physiologically, the body responds to a tissue insult, which can be physical, chemical, or biological in nature, by activating a dynamic network of cellular and molecular signals that coordinate the inflammatory response.^2^ Typical phases: 1. inflammatory, in which platelet aggregation and recruitment of inflammatory cells to the injured site occur; 2. proliferative, in which fibroblasts, keratinocytes, and endothelial cells migrate and proliferate, leading to re-epithelialization and the formation of granulation tissue; and 3. final phase of remodeling.^3^ These phases are regulated by a complex network of cytokines, growth factors, and cellular responses.^4^ Ultimately, these coordinated events restore skin architecture and function.^5^ Although this is a finely regulated process, if the balance between the various phases is altered due to disease or infection, the wound may heal very slowly or become chronic, resulting in serious clinical consequences such as infections, scarring, or poor tissue regeneration. Understanding the processes underlying WH is crucial for developing innovative therapeutic strategies that can optimize tissue regeneration and improve clinical outcomes in patients with acute or chronic wounds. For this reason, in recent years, a key element attracting growing interest has been natural products, particularly plant oils. They offer significant potential for promoting skin repair thanks to their antioxidant, anti-inflammatory, and regenerative properties.^6^ In particular, almond oil, especially the one obtained through unrefined cold pressing, is characterized by its notable content of tocopherols, monounsaturated fatty acids, and phytosterols.^7^ These bioactive constituents contribute to skin hydration, restore the epidermal barrier, and protect against oxidative processes induced by environmental factors. Cold pressing, compared to other extraction methods that use solvents or high temperatures, preserves the biochemical and nutritional properties of almond oil, reducing the environmental impact of the production process and promoting a circular economy.^8,9^ Almond oil is known for its emollient, anti-inflammatory, and skin barrier-supporting properties; several studies have observed that it improves skin hydration and quality, as well as in the prevention of pressure sores and the treatment of postsurgical scars.^1,10^ Furthermore, almond oil has emerged as an effective ingredient in skin care formulations, as it helps to strengthen the integrity of the stratum corneum and promotes its repair, improving the lipid ratio.^11^ However, evidence of the topical application of almond oil to wounds remains limited, requiring further investigation to confirm its efficacy. Accordingly, the present study investigated whether topical almond oil promotes WH and elucidated the molecular mechanisms underlying its antioxidant, anti-inflammatory, and regenerative effects, supporting its potential use in clinical and nutraceutical fields.

## 2 Materials and Methods

### 2.1 Animals

Male CD1 mice (25–30 g, Envigo, Milan, Italy) were housed in a controlled environment and given free access to food and water. All animal experiments were performed following protocols evaluated and approved by the Organismo Preposto al Benessere degli Animali (OPBA) of the University of Messina (ethics approval number: 1141/2024-PR). All procedures were conducted in accordance with applicable legislation for the protection of laboratory animals, including Italian Legislative Decree 26/2014 and the European Directive 2010/63/EU.

### 2.2 Nutrition Facts of Fresh Vintage Farms Almond oil

Almond oil was kindly provided by freshvintagefarms.com from the Almond Board of California. In short, almond oil is cold-pressed, resulting in artisanal and more nutritious oils. Nutritional information per 12.68 mL serving: 135 calories, 130 calories from fat, total fat 15 g (19%*), saturated fat 1 g (6%), trans fat 0 g, polyunsaturated fat 3 g, monounsaturated fat 10 g, cholesterol 0 mg (0%), sodium 0 mg (0%), total carbohydrate 0 g (0%), and protein 0 g (0%).

### 2.3 Experimental Wound Model

Mice received anesthesia with isoflurane before their back hair was cut, and the dorsum was prepared for wounding by applying a betadine scrub and 70% ethanol alternately three times. A sterile biopsy punch (KAI Corporation, Tokyo, Japan) was used to make two circular, full-thickness skin wounds on either side of the median line on the dorsum, each measuring 5 mm in depth.^2^ After 7 days, the mice were euthanized by cervical dislocation under anesthesia. The wound tissue was then divided into two parts: one was collected for histological analysis, and the other was collected for molecular analysis. Each wound site was digitally photographed at 0, 3, and 7 days, and the wound area was quantified using ImageJ software and expressed as the percentage of the original wound size over time.

### 2.4 Experimental Groups

The animals were randomly distributed into the following groups (*n = 6* for each group):

- Sham group: Mice were subjected to all procedures described above, and full-thickness excision wounds were not applied.
- WH: Mice were subjected to full-thickness excisional wounds as described above and received no topical treatment throughout the 7-day experimental period, serving as the injured control group.
- WH + almond oil: Animals were subjected to full-thickness excisional wounds as described above, and the wounds were treated once a day for 7 days with a topical application of 400 μL of almond oil administered with a micropipette.

The dose of almond oil (400 μL) was selected based on preliminary experiments conducted in our laboratory to determine the volume required for complete and uniform coverage of the wound area without dripping.

### 2.5 Histological Analysis

Sections of the wound area were stained with hematoxylin/eosin (H&E) and analyzed using a Leica DM6 microscope associated with Leica LAS X Navigator software (Leica Microsystems SpA, Milan, Italy).^12,13^ The histological score on a point scale from 0 to 4 was determined as previously described.^2^ Briefly, the scores were 0: absence of epithelial proliferation in >70% of the tissue; 1: poor epidermal organization in >60% of the tissue; 2: incomplete epidermal organization in >40% of the tissue; 3: moderate epithelial proliferation in >60% of the tissue; and 4: complete epidermal remodeling in >80% of the tissue.

### 2.6 Masson’s Trichrome Staining

Sections were also stained by Masson’s trichrome method for collagen detection.^2^ The relative density of blue-stained collagen after Masson’s trichrome staining was quantified in the wound area using ImageJ.

### 2.7 Immunohistochemical for Nitrotyrosine and Poly­(ADP-ribose polymerase) (PARP)

Immunohistochemical analysis was performed as previously described.^13^ Sections of the wound area were incubated with the primary antibodies: antinitrotyrosine antibody (Millipore, 1:200 in PBS, v/v), and antipoly­(ADP)-ribose (PAR) antibody (Santa Cruz Biotechnology (SCB), 1:500 in PBS, v/v). Samples were washed with PBS before being exposed to secondary antibodies for incubation.^14^ A biotin-conjugated goat antirabbit IgG and an avidin–biotin peroxidase complex were used to identify the specific labeling (Vector Laboratories, Burlingame, CA, USA).^15^ Using a Leica DM6 microscope (Leica Microsystems S.p.A., Milan, Italy), the stained sections were examined as per standard practice.^16,17^ The histogram profile is related to the positive-pixel intensity value obtained.

### 2.8 Evaluation of Cytokines

A colorimetric commercial ELISA kit was used to measure the levels of IL-1β, TNF-α, and IL-6 (R&D Systems, Minneapolis, MN, USA).

### 2.9 Western Blot Analysis

Western blot analysis was performed on skin samples as previously described.^18^ The following primary antibodies were used: anti-MMP-9 (1:500; SCB, sc-393859); anti-TGF-β (1:1000; Abcam, ab-9758); anti-VEGF (1:1000; SCB, sc-57496); anti-Nrf2 (1:100; SCB, sc-365949), anti-HO-1 (1:100; SCB, sc-136960); anti-SOD-2 (1:100, SCB, sc-137254); anti-NF-κB p65 (1:100; SCB, sc-841465); and anti-IkB-α (1:500; SCB, sc-1643), in phosphate-buffered saline, 5% *w*/*v* nonfat dried milk, and 0.1% Tween-20 at 4 °C overnight. Membranes were incubated with peroxidase-conjugated bovine antimouse IgG secondary antibody or peroxidase-conjugated goat antirabbit IgG (Jackson ImmunoResearch, West Grove, PA, USA; 1:2000) for 1 h at room temperature. Anti-β-actin (1:500; SCB, sc-8432) or antilamin A/C (1:500; Sigma-Aldrich) antibodies were used as controls. Protein expression was quantified by densitometry with Bio-Rad ChemiDocTM XRS+ software and normalized to housekeeping genes β-actin and lamin A/C as previously reported.^19^

### 2.10 Materials

Unless otherwise stated, all compounds were purchased from Sigma-Aldrich. All solutions used for in vivo infusions were prepared using nonpyrogenic saline (0.9% NaCl; Baxter Healthcare Ltd., Thetford, Norfolk, UK).

### 2.11 Statistical Evaluation

An a priori power analysis was conducted using G*Power software to estimate the minimum sample size required for each experimental group. The analysis was performed before the experimental procedures to ensure an adequate number of animals for the study. All values are expressed as mean ± standard error of the mean (SEM) of *N* observations. The images shown are representative of at least 3 experiments performed on different experimental days on tissue sections collected from all animals in each group. For in vivo studies, *N* represents the number of animals used. The results were analyzed by one-way ANOVA followed by a Bonferroni post hoc test for multiple comparisons. A *P* value less than 0.05 was considered significant.

## 3 Results

### 3.1 Effects of Topical Application of Almond Oil on Wound Closure and Histological Tissue Damage

As shown in Figure 1A, on day 0, we created full-thickness excisional wounds with a 5 mm biopsy punch. Almond oil was administered topically (400 μL) to the wound site for 7 days to test its potential therapeutic effects on the healing process. As shown by the wound images and the wound closure percentage graph (Figure 1A,B), we observed that, compared to the control, daily almond oil treatment significantly improved the WH process from day 3 to day 7 in terms of the speed and percentage of wound closure. Furthermore, to observe the effect of almond oil on WH-induced tissue changes, hematoxylin and eosin (H&E) staining was performed (Figure 1C,D,E, and relative histological analysis, Figure 1F). H&E staining showed a compromised wound characterized by extensive tissue damage, a large number of neutrophils and fibroblasts in edematous tissue, and limited cutaneous and epidermal organization in the WH (Figure 1D, and relative histological analysis, Figure 1F), compared to tissue from the sham group (Figure 1C, and relative histological analysis, Figure 1F). Topical administration of almond oil (Figure 1E and relative histological analysis, Figure 1F) resulted in improvements in wound closure, granulation, and re-epithelialization, all of which contributed to near-complete restoration of the epidermis in this group.

**Figure 1:**
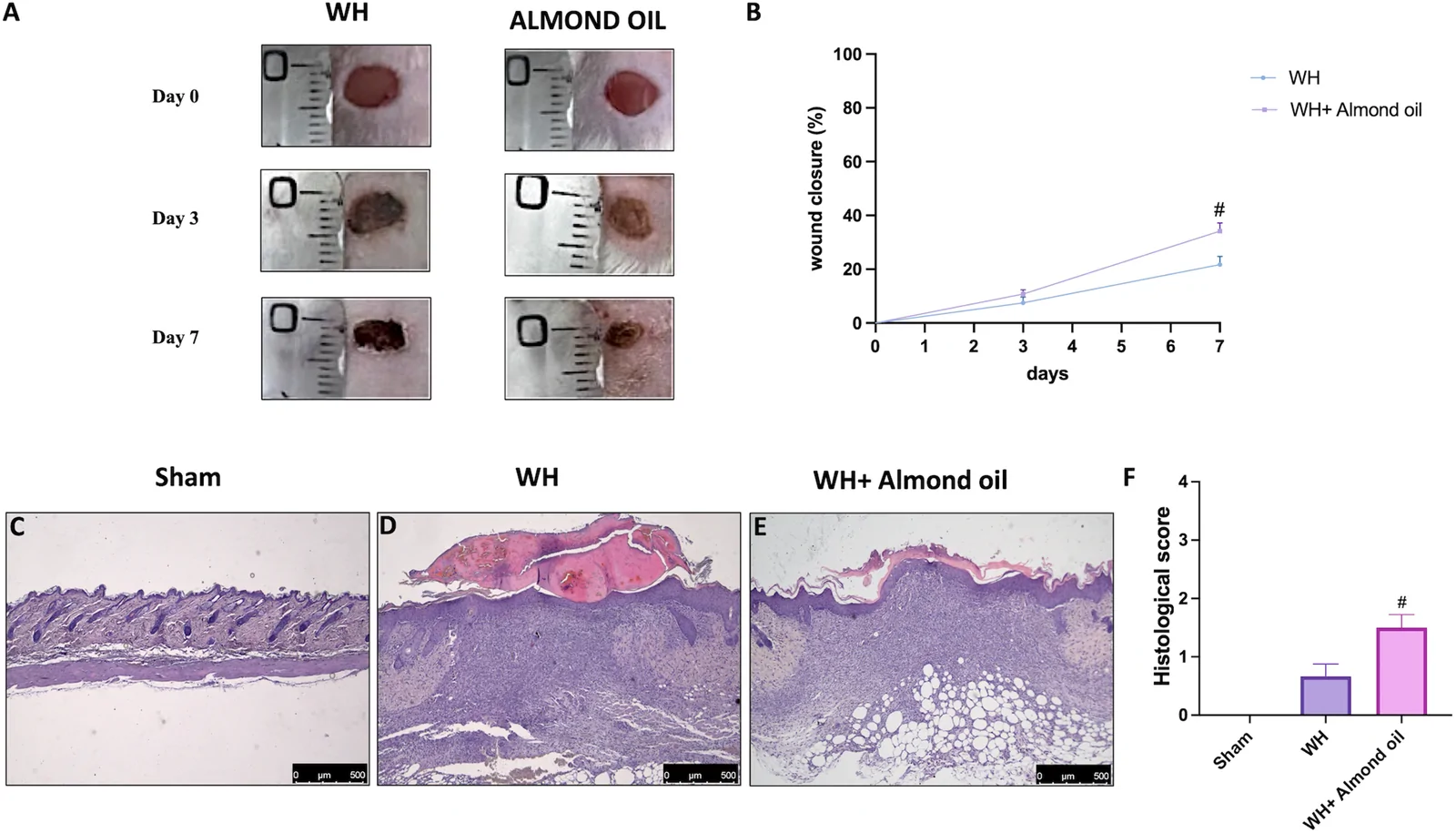
Effect of topical application of almond oil on WH rate and histological damage in a murine full-thickness excisional wound model. (A) Representative picture of wounds at the different time points. (B) The graph shows the results expressed as the percentage of original wound size over time. (C–F) H&E staining of wound granulation tissue at 7 days after induction and relative histological score graph. Data are presented as means ± SEM of 6 mice for each group. ^#^ *p* < 0.05 vs WH.

### 3.2 Effect of Topical Application of Almond Oil on Collagen Fibers and the Expression of MMP-9, TGF-β, and VEGF in Wounded Skin

To evaluate extracellular matrix remodeling in the dermis, Masson’s trichrome staining was performed. The staining revealed the presence and organization of both fine and coarse collagen fibers in the wounded skin tissue (Figure 2A–C; graph D). In the control group, normal collagen deposition was observed (Figure 2A), whereas the WH group showed a marked reduction in collagen content 7 days after wound induction (Figure 2B). Significantly, the WH + almond oil group exhibited enhanced collagen fiber synthesis and improved organization around the wound area (Figure 2C), indicating a stimulatory effect of topical almond oil treatment. WH is closely associated with fibrosis and neovascularization, both of which are regulated by various signaling molecules. Among them, MMPs play a critical role in tissue remodeling. To further investigate the molecular mechanisms involved, we analyzed the expression of MMP-9, TGF-β, and VEGF by Western blot. As shown in Figure 2E (densitometric analysis in Figure 2E1), the expression of MMP-9 was significantly elevated in wounded tissue but was notably reduced following treatment with almond oil, suggesting its inhibitory effect on wound-induced MMP-9 upregulation. Regarding TGF-β expression (Figure 2F; densitometric analysis in Figure 2F1), a high level was detected in the sham group, while it was reduced in the WH group. Topical almond oil treatment significantly restored TGF-β expression to levels comparable to the control. Finally, VEGF expression (Figure 2G; densitometric analysis in Figure 2G1) was significantly increased in the WH group and further upregulated in the WH + almond oil group, confirming the pro-angiogenic effect of the treatment.

**Figure 2:**
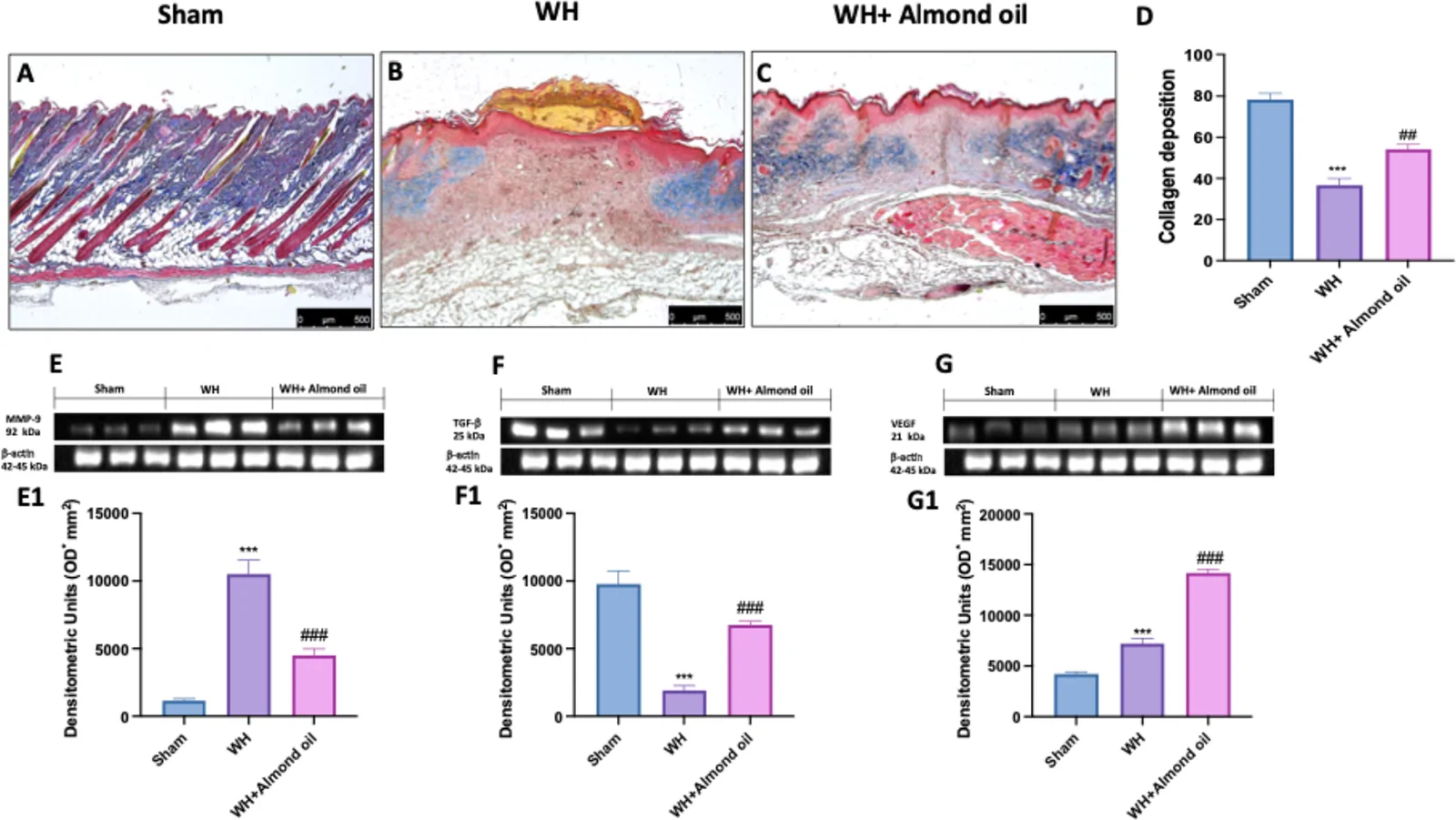
Effect of topical almond oil treatment on collagen deposition (Masson’s trichrome staining) and expression levels of MMP-9, TGF-β, and VEGF, 7 days after injury. (A–C and relative collagen quantification in graph D) Masson’s trichrome staining shows collagen fibers in the control group (A), reduced fibers in the WH group (B), and enhanced deposition in the WH + almond oil group (C). (E–G): Western blot analysis of MMP-9 (E), TGF-β (F), and VEGF (G) expression levels. (E1–G1): Corresponding densitometric analyses. Exposed is a blot of lysates (6 animals/group) with a densitometric analysis for all animals. Data are expressed as means ± SEM of six animals for each group. *** *p* < 0.001 versus sham. ^##^ *p* < 0.01 vs WH. ^###^ *p* < 0.001 vs WH.

### 3.3 Effect of Topical Application of Almond Oil on Nitrotyrosine and PARP Expressions

The expression of nitrotyrosine, a specific marker of nitrosative stress, and PARP, a marker of DNA degradation, was analyzed by immunohistochemical staining. Skin tissue sections from sham-operated mice showed no evidence of nitrotyrosine staining (Figure 3A, A1), whereas sections from WH mice showed robust positive staining for nitrotyrosine (Figure 3B, B1). Furthermore, increased positive staining for PARP was also observed in tissues from WH mice (Figure 4B, B1) compared to the sham group (Figure 4A, A1). Topical treatment with 400 μL of almond oil significantly reduced positive staining for nitrotyrosine and PARP in all tissues (Figure 3C, C1 and Figure 4C, C1, and see related graphs in Figure 3D and Figure 4D).

**Figure 3:**
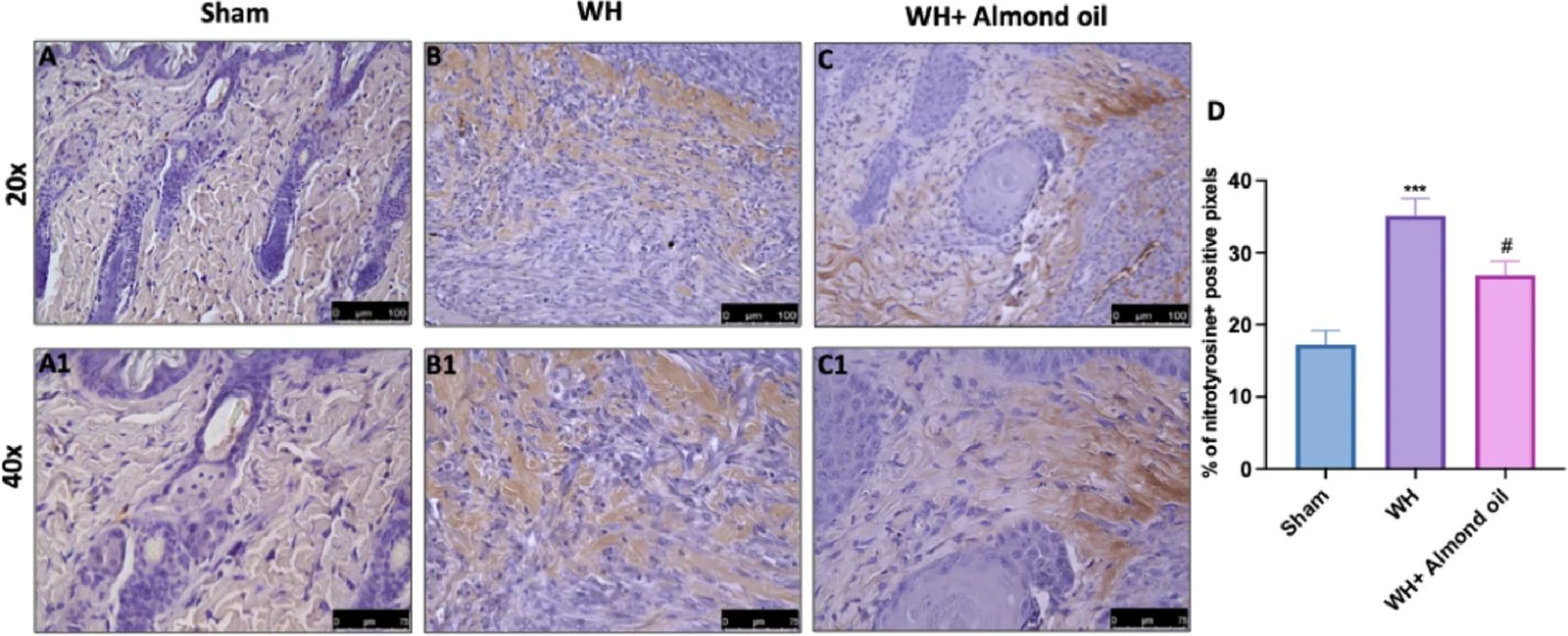
Effects of topical almond oil treatment on nitrotyrosine in skin 7 days after wounding. Immunohistochemical analysis showed positive staining for nitrotyrosine in the WH group compared to the sham group (B, B1, A, A1). Reduced nitrotyrosine expression was observed in skin from the WH + almond oil group 7 days after wounding (C, C1). The results are expressed as % of positive pixels (D). Data are presented as means ± SEM of 6 mice for each group. *** *p* < 0.001 vs sham. # *p* < 0.05 vs WH.

**Figure 4:**
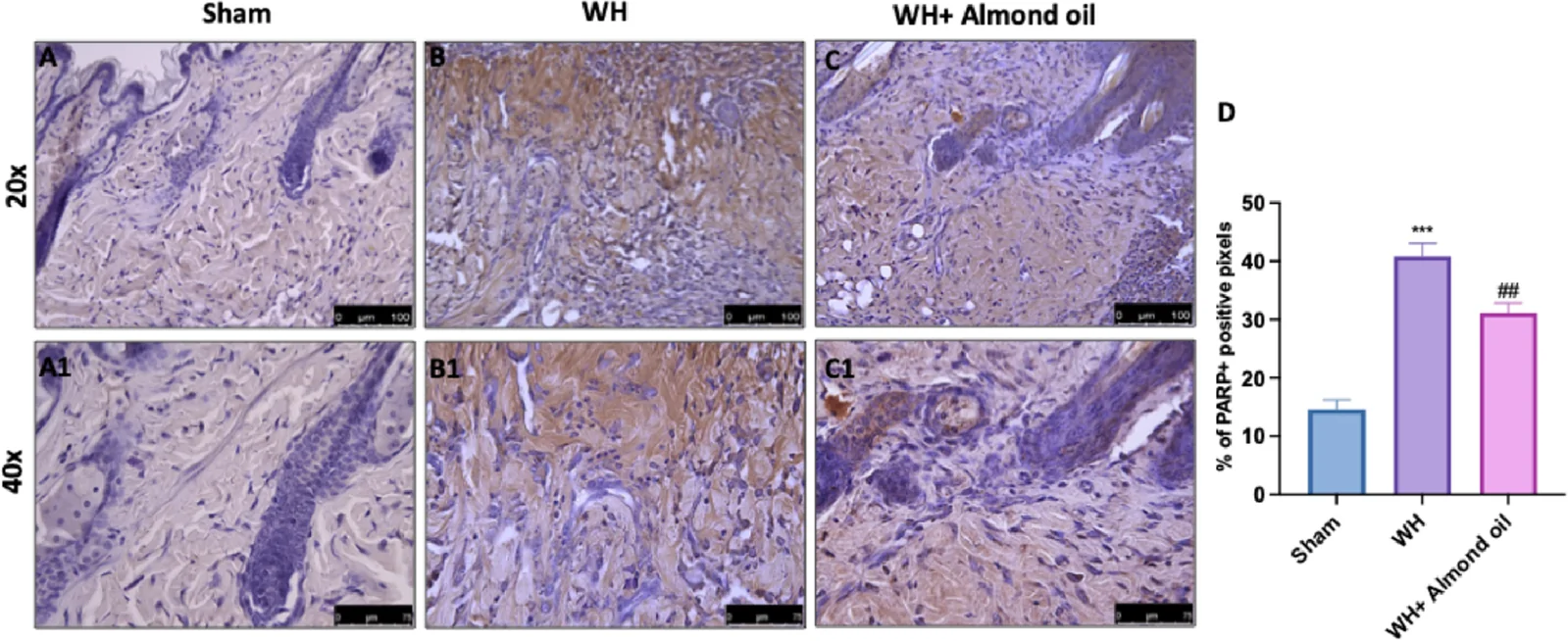
Effects of topical almond oil treatment on PARP in skin 7 days after wounding. Immunohistochemical analysis showed positive staining for PARP in the WH group compared to the sham group (B, B1, A, A1). Reduced PARP expression was observed in skin from the WH + almond oil group 7 days after wounding (C, C1). The results are expressed as % of positive pixels (D). Data are presented as means ± SEM of 6 mice for each group. *** *p* < 0.001 vs sham. ## *p* < 0.01 vs WH.

### 3.4 Effect of Topical Almond Oil Application on Inflammation Induced by Full-Thickness Excisional Wounds

To evaluate the anti-inflammatory activity of topical application of almond oil to the wound, we analyzed the levels of IL-1β (Figure 5A), TNF-α (Figure 5B), and IL-6 (Figure 5C). Levels of these cytokines were significantly increased in the WH group compared to sham-operated mice. In contrast, cytokine release was significantly reduced in mice treated with almond oil. An increase in NF-κB nuclear translocation was found after wound induction (Figure 5E) along with a reduced expression of its inhibitor IκB-α (Figure 5D, see densitometric analysis Figure 5E1, D1). Treatment with almond oil was able to reduce NF-κB expression levels (Figure 5E and densitometric analysis Figure 5E1) and restore IκB-α expression (Figure 5D, see Figure 5D1).

**Figure 5:**
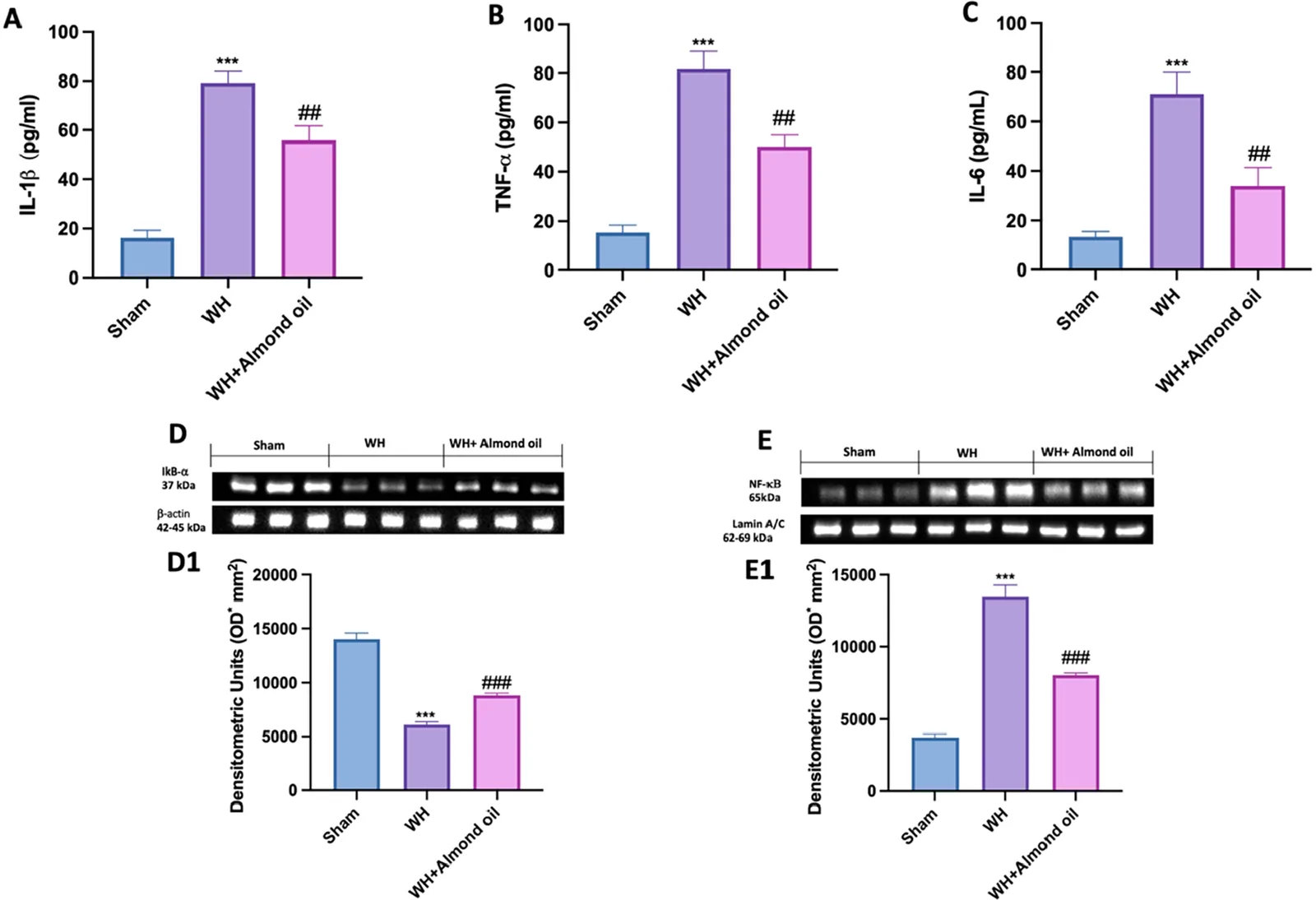
Effects of topical application of almond oil on the levels of the cytokines and on inflammation. Measurement of IL-1β (A), TNF-α (B), and IL-6 (C). Representative Western blots showing the effects of almond oil on: (D) Ikb-α expression, (E) NF-κB expression at 7 days postinjury. Almond oil treatment increased Ikb-α levels (D) and reduced NF-κB expression (E) in the WH + almond oil group compared to the WH group. Exposed is a blot of lysates (6 animals/group) with a densitometric analysis (D1, E1) for all animals. Data are expressed as means ± SEM of six animals for each group. ***p < 0.001 vs sham. ^##^ *p* < 0.01 vs WH, ^###^ *p* < 0.001 vs WH.

### 3.5 Effect of Topical Application of Almond Oil on Oxidative Stress

To further investigate whether almond oil may act by interacting with signaling pathways such as Nrf2/HO-1, Western blot analyses for Nrf2/HO-1 and SOD-2 were also performed on skin tissues following wound induction. A reduction in nuclear translocation of Nrf2 was found in the WH group, compared to the sham group, after wound induction (Figure 6A, see densitometric analysis Figure 6A1), while treatment with almond oil increased the expression of Nrf2 compared to the WH group (Figure 6A, see densitometric analysis Figure 6A1). At the same time, Western blot analysis showed that after treatment with almond oil, the expression of HO-1 protein and SOD-2 significantly increased compared to the WH group (Figure 6B,C, see densitometric analysis Figure 6B1 and C1), indicating an increase in the antioxidant activity of the bioactive molecules present in almond oil.

**Figure 6:**
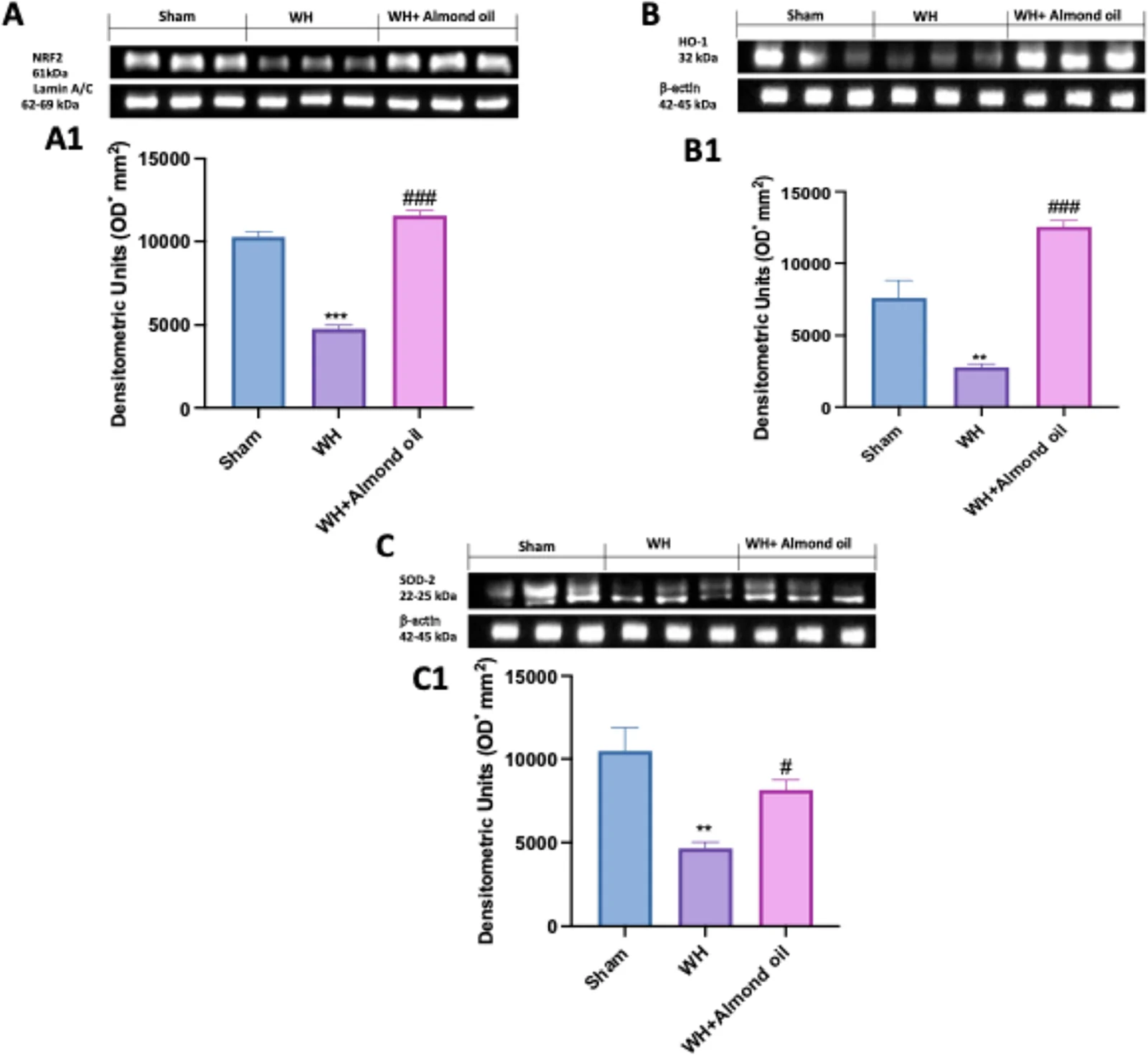
Effects of topical application of almond oil on oxidative stress. Representative Western blots showing the effects of almond oil on: (A) Nrf2 expression, (B) HO-1 expression, and (C) SOD-2 expression at 7 days postinjury. Almond oil treatment increased Nrf2 (A), HO-1 (B), and SOD-2 expression (C) in the WH + almond oil group compared to the WH group. Exposed is a blot of lysates (6 animals/group) with a densitometric analysis (A1, B1, and C1) for all animals. Data are expressed as mean ± SEM of six animals for each group. *** *p* < 0.001 vs sham **p < 0.01 vs sham. ^#^ *p* < 0.05 vs WH. ^###^ *p* < 0.001 vs WH.

## 4 Discussion

WH is a fundamental process for the body and for this reason it is necessary that it can restore the integrity of the skin in the best possible way.^20^ The skin is essential for maintaining physiological homeostasis, and the WH process is divided into four phases: hemostasis, inflammation, proliferation, and remodeling.^21^ Furthermore, these phases include contraction, granulation, epithelialization, and collagen deposition.^22^ WH involves a complex and tightly regulated series of molecular events orchestrated by a variety of cell types recruited to the site of injury, including keratinocytes, fibroblasts, neutrophils, and macrophages.^23–25^ Compromised WH is a major concern for hospitalization, particularly in conditions where wounds do not heal properly, and therefore finding alternative therapies for restoring skin integrity is of paramount importance.^6,26^ Among naturally occurring substances, cold-pressed almond oil stands out for its healing and regenerative properties. This process preserves the essential fatty acids, fat-soluble vitamins, and biologically active compounds responsible for its benefits.^7,9,11^ This study explored the physiological and therapeutic potential of almond oil applied topically to full-thickness excisional wounds in mice, with particular attention to the mechanisms underlying its regenerative, antioxidant, and anti-inflammatory effects. Our results, obtained through histological analysis and Masson’s trichrome staining, showed that topical application of almond oil accelerated wound closure from the third to the seventh day after injury; the improvement in the healing response on the seventh day was further confirmed by better tissue organization, re-epithelialization, granulation tissue formation, and collagen deposition. These observations were confirmed by the analysis of biomolecular markers: MMP-9, TGF-β, and VEGF. MMPs are a family of zinc-dependent endopeptidases that degrade nearly all proteins in the extracellular matrix and the basement membrane at neutral pH. Their main functions include the degradation of damaged extracellular matrix during the inflammatory phase, the breakdown of the capillary basement membrane to promote angiogenesis and cell migration during the proliferation phase, and tissue remodeling. Our study demonstrated that topical treatment with almond oil reduced the wound-associated overexpression of MMP-9, suggesting a more favorable extracellular matrix remodeling environment. As previously mentioned, TGF-β is another important marker, involved in all phases of WH: inflammation, angiogenesis, fibroblast proliferation, collagen synthesis and deposition, as well as the restoration of new extracellular matrix.^3^ As shown by Western blot analysis, almond oil was able to restore TGF-β levels to those of the sham group, a significant result when compared to the expression levels in the WH group, which showed a reduction in TGF-β 7 days after skin excision, associated with its role in repairing the extracellular matrix during tissue regeneration. TGF-β is, in turn, known to recruit VEGF-generating hematopoietic effector cells to stimulate angiogenesis.^15,27^ VEGF plays a central role in inducing angiogenesis based on its ability to stimulate the expression of proteases that digest extracellular matrix components that block angiogenesis, stimulate endothelial cell proliferation, and prevent their apoptosis.^28^ In accord with this, the elevated VEGF expression observed after almond oil treatment suggests increased angiogenic signaling, which may contribute to the improved tissue repair observed in the injured area. These mechanistic findings provide a plausible biological basis that may help explain the beneficial effects of almond oil reported in previous human studies. In a controlled trial in critically ill patients, daily topical application of almond oil markedly reduced the incidence of pressure injuries compared with paraffin or standard care only and delayed their onset when they did occur.^10^ Similarly, in patients with split-thickness skin graft donor sites, almond oil showed outcomes comparable to those of a specialized regeneration oil blend in terms of scar quality, itching, and discoloration, supporting its potential use in skin and scar care.^1^ These data demonstrate that the benefits of almond oil extend beyond simple wound closure, involving angiogenic signaling and extracellular matrix remodeling, critical components of effective tissue repair. To assess oxidative and nitrosative stress, we measured the expression of nitrotyrosine and PARP. Nitrotyrosine is a biomarker of oxidative stress due to the nitration of protein-bound and free tyrosine residues by reactive peroxynitrite molecules.^29^ Immunohistochemical analysis for nitrotyrosine showed marked positivity in the WH group, indicating nitrosative damage to proteins and lipids at the wound site 7 days after injury. Peroxynitrite is also a potent inducer of DNA damage and is responsible for the concomitant increase in PARP expression observed in the same tissues. Activation of this enzyme has been shown to lead to hyperactivation of PARP, which impairs keratinocyte migration, increases the production of inflammatory mediators, and limits angiogenesis, all of which delay wound closure.^30^ Topical treatment with almond oil significantly reduced both nitrotyrosine and PARP positivity, suggesting a reduction in nitrosative and cellular stress associated with wound injury. This effect appears to be associated with the activation of the Nrf2/HO-1/SOD-2 axis induced by almond oil. Nrf2, the main regulator of the antioxidant response, once activated, translocates to the nucleus and induces a series of cytoprotective enzymes, including HO-1 and SOD-2. The reduced expression of Nrf2 in the WH group is indicative of deficiencies in the antioxidant defense system, which has become insufficient to counteract oxidative/nitrosative damage. Its restoration by almond oil, along with the simultaneous increase in HO-1 and SOD-2, is consistent with a restoration of the body’s antioxidant defenses. Inflammatory regulation represents another important component of the observed response.^15,31,32^ NF-κB is a key transcription factor involved in the regulation of a wide range of genes and pathways implicated in inflammation. Almond oil treatment led to reduced levels of IL-1β, TNF-α, and IL-6, reduced expression of NF-κB, and increased expression of IκB-α, supporting the attenuation of excessive pro-inflammatory signaling. Instead of suggesting the total inhibition of inflammation, the present findings are compatible with the modulation of the inflammatory milieu that may favor the transition to the next proliferative and remodeling stages of WH.

Overall, our results demonstrate that topical application of almond oil accelerates WH through a multifaceted mechanism involving anti-inflammatory action via inhibition of NF-κB, reduction of oxidative and nitrosative stress via activation of Nrf2/HO-1/SOD-2, promotion of collagen deposition and tissue regeneration via regulation of MMP-9 and TGF-β, and stimulation of angiogenesis via increased VEGF expression. In addition to the pathways examined here, apoptosis is another important process involved in WH, contributing to the removal of cells during the transition from the proliferative to the remodeling phase.^33^ The decrease in nitrotyrosine and PARP expression and the activation of the Nrf2/HO-1/SOD-2 pathway following almond oil therapy suggest a reduction in cellular stress, although the present study did not directly assess apoptosis. Therefore, the probable significance of almond oil in the control of apoptosis should be studied. Future research concentrating on apoptotic indicators such as cleaved caspase-3, Bax/Bcl-2 ratio, and TUNEL staining could assist in explaining the significance of this process in the regeneration effects reported after almond oil administration. This study has some limitations that should be considered when interpreting its translational implications. First, the experimental model consisted of acute full-thickness excisional wounds in mice with a 7-day follow-up period, which primarily captures the early inflammatory and proliferative phases of WH and does not allow assessment of long-term outcomes such as scar maturation or tissue remodeling.^34^ Second, wound closure in mice is driven largely by contraction of the panniculus carnosus, a subcutaneous muscle layer that is absent in human skin, whereas human wounds close primarily through re-epithelialization and granulation tissue formation.^35,36^ This anatomical difference may limit the direct translation of healing kinetics and some mechanisms observed in the present model to human wounds. Therefore, validation in models that more closely reproduce human wound closure, such as splinted wound models, together with longer-term and clinical studies, will be important to further establish the translational potential of almond oil. Although the study has limitations, it provides integrated histological and molecular evidence demonstrating that topically applied almond oil promotes WH through coordinated modulation of inflammatory processes, oxidative/nitrosative stress, extracellular matrix remodeling, and angiogenic pathways, providing a mechanistic basis for its potential use as a natural topical approach in wound management.

## 5 Conclusion

In conclusion, in this study, we observed that topical application of almond oil can modulate and accelerate skin WH in mice, inducing the expression of numerous factors involved in different phases of the healing process. These results offer experimental proof of almond oil’s translational potential as a topical medication for the treatment of skin wounds, which is accessible, safe, and long-lasting. Its significance as a supplementary or alternative strategy in clinical wound care is highlighted by its capacity to modify important molecular pathways involved in inflammation, oxidative stress, and tissue regeneration. To confirm its effectiveness, improve formulation, and assess long-term safety in human applications, more translational and clinical research is necessary.

## Abbreviations

WH: wound healing
MMP-9: matrix metalloproteinase-9
TGF-β: transforming growth factor-β
VEGF: vascular endothelial growth factor
Nrf2: nuclear factor erythroid 2-related factor 2
HO-1: heme oxygenase-1
SOD-2: superoxide dismutase 2
NF-κB: nuclear factor kappa B
IκB-α: nuclear factor of kappa light polypeptide gene enhancer in B-cells inhibitor, α
PARP: poly­(ADP-ribose) polymerase
ROS: reactive oxygen species
IL-1β: interleukin-1 β
IL-6: interleukin-6
TNF-α: tumor necrosis factor-α
